# In Sections or Otherwise

**Published:** 1904-12-15

**Authors:** J. D. Patterson


### IN SECTIONS OR OTHERWISE.

BY J. D. PATTERSON, D.D.S.

It has been the opinion of the writer—since the movement towards separate and simultaneous section meetings for the reading of papers and their discussion in our national bodies has gained force—that the old plan long in vogue in the American Dental Association was the wisest, and added most to the interest and secured the larger attendance at those meetings. The recent experience at the Congress at St. Louis has only confirmed that opinion. If, in the profession of dentistry, section meetings were demanded, surely the demand, and the necessity for the demand, Would have presented at a World's Dental Congress Instead of this, the experience at the Congress has only shown to the observing that section meetings have signally failed, and that a large majority are opposed to them and desire a less complicated method to arouse and concentrate the interest of all.

The National Dental Association, at Asheville in 1903, adopted the new rules, providing for separate meetings in three sections; and while it may be said that the new plan has had no trial, yet it is fair to judge of the outcome in the light of recent experience, and that the plan is faulty so far as our profession is concerned. There *has* been, and we believe *always will be*, a paucity of interest and attendance in some of such section meetings, and a confusion of wandering interest as to which is the most desirable to attend, so that a positive disadvantage will always be manifest.

Dr. Ottolengui, in *Items of Interest*, correctly states that in the medical profession, with its long array of specialties, it is appropriate and necessary to have those specialists meet in separate rooms and hold separate sessions; but in our profession specialties are *in embryo*, and only one, that of orthodontia, can at all be considered as a specialty. Even orthodontia can scarcely be so considered when all dental practitioners practice the art, and only less than a score in the whole country of the United States are exclusively engaged in the work.

Three sections, as now provided for in our National Association, will, we believe, present the objections to separate section meetings just as prominently as if there were a greater number; and if the old rule in the American Association had been continued and *properly lived up to*, there would have been no need to ape the methods of medical organizations with large membership, in this country or any other. In our opinion, the trouble is that the old methods were so poorly lived up to that the new ones have been broached. If section officers had but done their duty, and in the afternoon of the first day of meeting insisted upon the presentation of all papers, and voted without discussion as to their merits as to whether they should be read before the main body or not; and if the presiding officer of the main body, when such papers were presented, had promptly and positively controlled the proceedings, and cut out all the fool nonsense that presiding officers are disposed to admit—there never would have been any movement for distinct section meetings for the presentation and discussion of papers, and it would have been found in all cases—as it was in some—that there was plenty of time to hear all acceptable papers and the discussion upon them. At the very meeting in Asheville where the separate section meeting rules were adopted, the time was frittered away and the association more than once adjourned because there were no more papers to be read.

This brings us to the gist of these remarks, and it is this: At a dental meeting, let those be selected to arrange the program and to preside who think more of their profession and the scientific success of the meeting than of their after-dinner coffee; men who will *demand* that sessions open promptly, brush aside extraneous interests, and confine the association to the legitimate business for which it was created. Then we would hear less of wondering what had come over the spirit of our National Association, and less of the talk for separate sectional meetings.—Editorial, *Western Journal.*

